# Locked fracture dislocations of the proximal humerus: postoperative results and a proposed modification of the classification

**DOI:** 10.1007/s00590-021-03022-z

**Published:** 2021-06-05

**Authors:** Jonas Schmalzl, Annika Graf, Fabian Gilbert, Michael Kimmeyer, Christian Gerhardt, Lars-Johannes Lehmann

**Affiliations:** 1 Department of Traumatology, Hand, Plastic and Reconstructive Surgery, Julius-Maximilians-University Wuerzburg, University Hospital Wuerzburg, Josef-Schneider-Str. 2, 97080 Wuerzburg, Germany; 2 Department of Traumatology and Hand Surgery, St. Vincentius Clinic, Teaching Hospital Albert-Ludwigs-University Freiburg, Karlsruhe, Germany; 3 Medical Faculty Mannheim, Karls-Ruprecht-University Heidelberg, Mannheim, Germany

**Keywords:** Fracture sequelae shoulder, Shoulder arthroplasty, Locked shoulder dislocation, Bone defect, Pectoralis major transfer, Glenoid bone grafting

## Abstract

**Background:**

Locked dislocations of the glenohumeral joint are disabling and often painful conditions and the treatment is challenging. This study evaluates the functional outcome and the different prosthetic treatment options for chronic locked dislocations of the glenohumeral joint and a subclassification is proposed.

**Methods:**

In this single-center retrospective case series, all patients with a chronic locked dislocation treated surgically during a four-year period were analyzed. Constant score (CS), Quick Disabilities of Shoulder and Hand Score (DASH), patient satisfaction (subjective shoulder value (SSV)), revision rate and glenoid notching were analyzed.

**Results:**

26 patients presented a chronic locked dislocation of the glenohumeral joint. 16 patients (62%) with a mean age of 75 [61–83] years were available for follow-up at 24 ± 18 months. CS improved significantly from 10 ± 6 points to 58 ± 21 points (*p* < 0.0001). At the final follow-up, the mean DASH was 27 ± 23 and the mean SSV was 58 ± 23 points. The complication rate was 19% and the revision rate was 6%; implant survival was 94%. Scapular notching occurred in 2 (13%) cases (all grade 1).

**Conclusion:**

With good preoperative planning and by using the adequate surgical technique, good clinical short-term results with a low revision rate can be achieved. The authors suggest extending the Boileau classification for fracture sequelae type 2 and recommend using a modified classification to facilitate the choice of treatment as the suggested classification system includes locked posterior and anterior dislocations with and without glenoid bone loss.

**Level of evidence:**

IV.

## Introduction

Chronic locked dislocation of the glenohumeral joint represents a rare shoulder pathology and is usually associated with previous trauma [1, 2]. Boileau et al. [3, 4] first classified this pathology among his classification for fracture sequelae of the proximal humerus. Chronic malposition of the humeral head can lead to soft tissue damage as well as bone loss of the glenoid or proximal humerus. Up to date, several treatment options such as humeral osteotomy, hemiarthroplasty (HA), anatomical total shoulder arthroplasty (TSA) or reverse shoulder arthroplasty (RSA) have been suggested [2, 3, 5, 6]. However, the complication rates are reported to be as high as 45% and the long-term survival rates are reported to be worse [1, 7]. For TSA recurrent instability and glenoid loosening have been described, whereas the main problem for RSA is reported to be the fixation of the glenoid component [2, 6]. Glenoid bone deficiency may compromise component fixation or sometimes even impede placement of a glenoid component at all [8]. In RSA, autologous bone grafting of glenoid defects supported by cementless baseplate fixation is a feasible procedure and recent literature indicates promising results [9, 10]. However, little is known about the limits of asymmetric glenoid bone loss reconstruction in cases of locked fracture dislocations of the proximal humerus. These cases might additionally be compromised by unbalanced loads due to soft tissue adaptations such as scarring, heterotopic ossification or tendon degeneration.

**Fig. 1:**
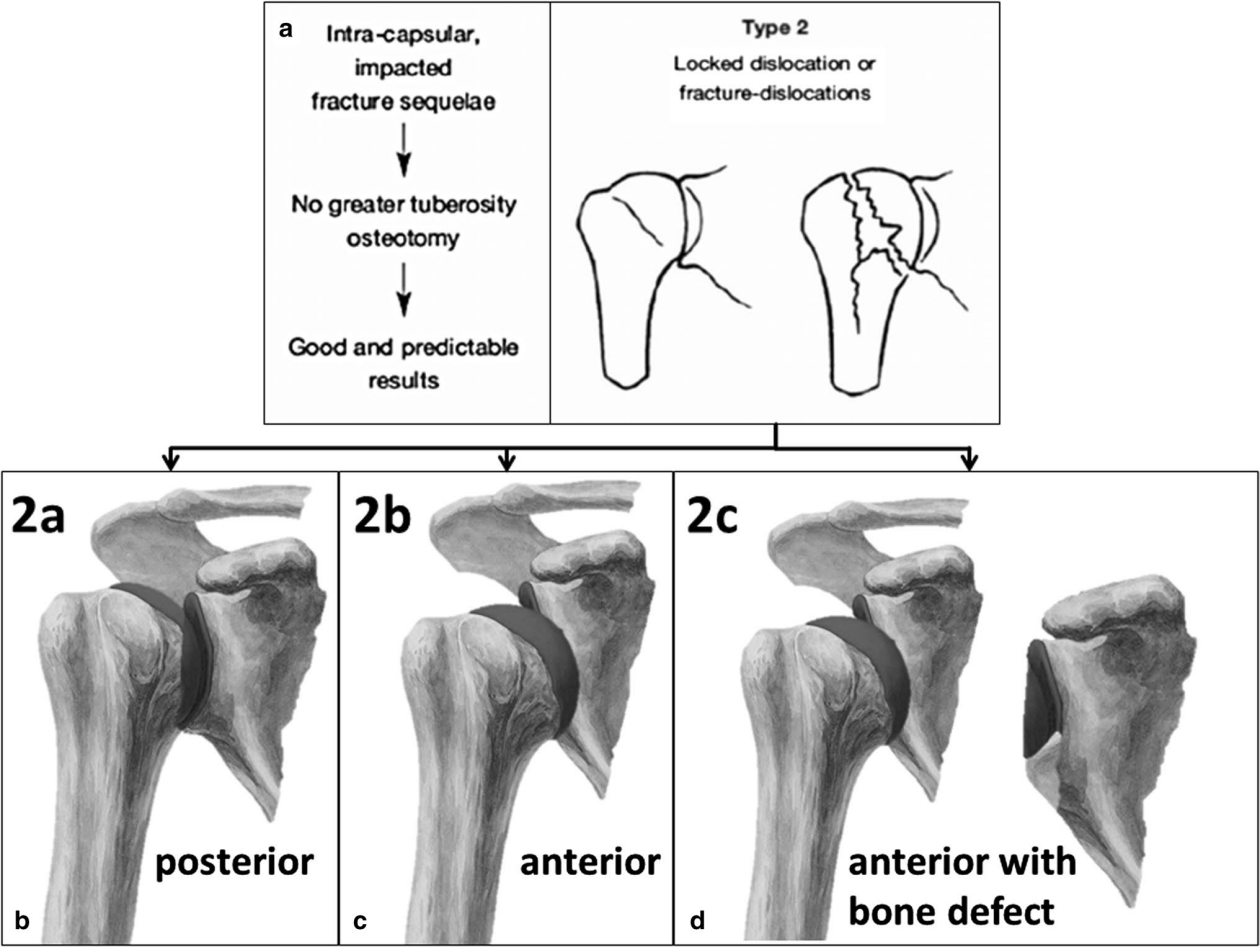
Subclassification of proximal humeral fracture sequelae type 2 according to Boileau (**a**). Type 2a lesions (**b**) are defined as locked posterior dislocations and can be treated with hemiarthroplasty or total shoulder arthroplasty. Type 2b lesions (**c**) represent locked chronic anterior dislocations and should be treated with reverse shoulder arthroplasty. Type 2c lesions (**d**) are defined as locked chronic anterior dislocations with glenoid bone loss and a treatment option is autologous bone grafting of the glenoid, implantation of a reverse prosthesis and pectoralis major tendon transfer for soft tissue balancing

The purpose of this study was to report our treatment results of locked fracture dislocations of the proximal humerus and to propose a new subclassification system dependent on direction of the dislocation and glenoid bone loss as shown in Fig. 1. The hypothesis was that the right choice of treatment would lead to reproducible and satisfying clinical results.

## Materials and methods

### Study design

This was a single-center retrospective case series. All consecutive patients with a FS type 2 according to Boileau [3] treated between 2014 and 2018 were included. Exclusion criteria were severe neurological comorbidities and noncompliance with the postoperative rehabilitation protocol.

### Compliance with ethical standards

Institutional review board approval was obtained prior to commencing the study. All patients signed informed consent and gave their approval for the use of clinical and radiographic data for scientific purposes. The conducted experiments respect the ethical standards in the Helsinki Declaration of 1975, as revised in 2000, as well as the national law.

Preoperative X-rays in 2 planes (anterior-posterior (AP) and Y-view) and a computed tomography (CT) scan of the affected side were obtained. All patients underwent surgery in beach chair position under general or regional anesthesia. Surgery was performed by one single surgeon (LL). A deltopectoral approach was performed in all cases.

### Boileau classification

Boileau et al. first classified fracture sequelase of the proximal humerus in 2001. This classification differentiates between intracapsular/impacted fracture sequelae (associated with both cephalic collapse or necrosis [type 1] and chronic dislocation or fracture-dislocation [type 2]), and extracapsular/disimpacted fracture sequelae (associated with both surgical neck nonunions [type 3] and severe tuberosity malunions [type 4]).

### Proposed subclassification

We suggest the subclassification of FS type 2 in three different subtypes. Type 2a—chronic locked posterior dislocations (normally without glenoid defect. Type 2b—chronic locked anterior dislocations without glenoid bone loss. Type 2c—chronic locked anterior dislocations with glenoid bone loss.

### Surgical technique for subtype 2a

For type 2a lesions of our suggested modified classification (Fig. 1), either a stemless hemiprosthesis or a stemless total shoulder prosthesis with a cemented keeled polyethylene glenoid component (dependent on the glenoid condition) was implanted (Eclipse; Arthrex, Naples, USA) according to the manufacturer’s instruction. Glenoid replacement was only performed in cases of osteoarthritis or large osteochondral defects of the glenoid fossa.

### Surgical technique for subtype 2b

For type 2b lesions of our suggested modified classification (Fig. 1), a cementless reverse total shoulder prosthesis with 135° humeral inclination and with + 4 mm glenosphere lateralization was implanted (Univers Revers; Arthrex, Naples, USA) according to the manufacturer’s instruction. If possible, the subscapularis tendon was reattached after implantation of the prosthesis.

### Surgical technique for subtype 2c

**Fig. 2:**
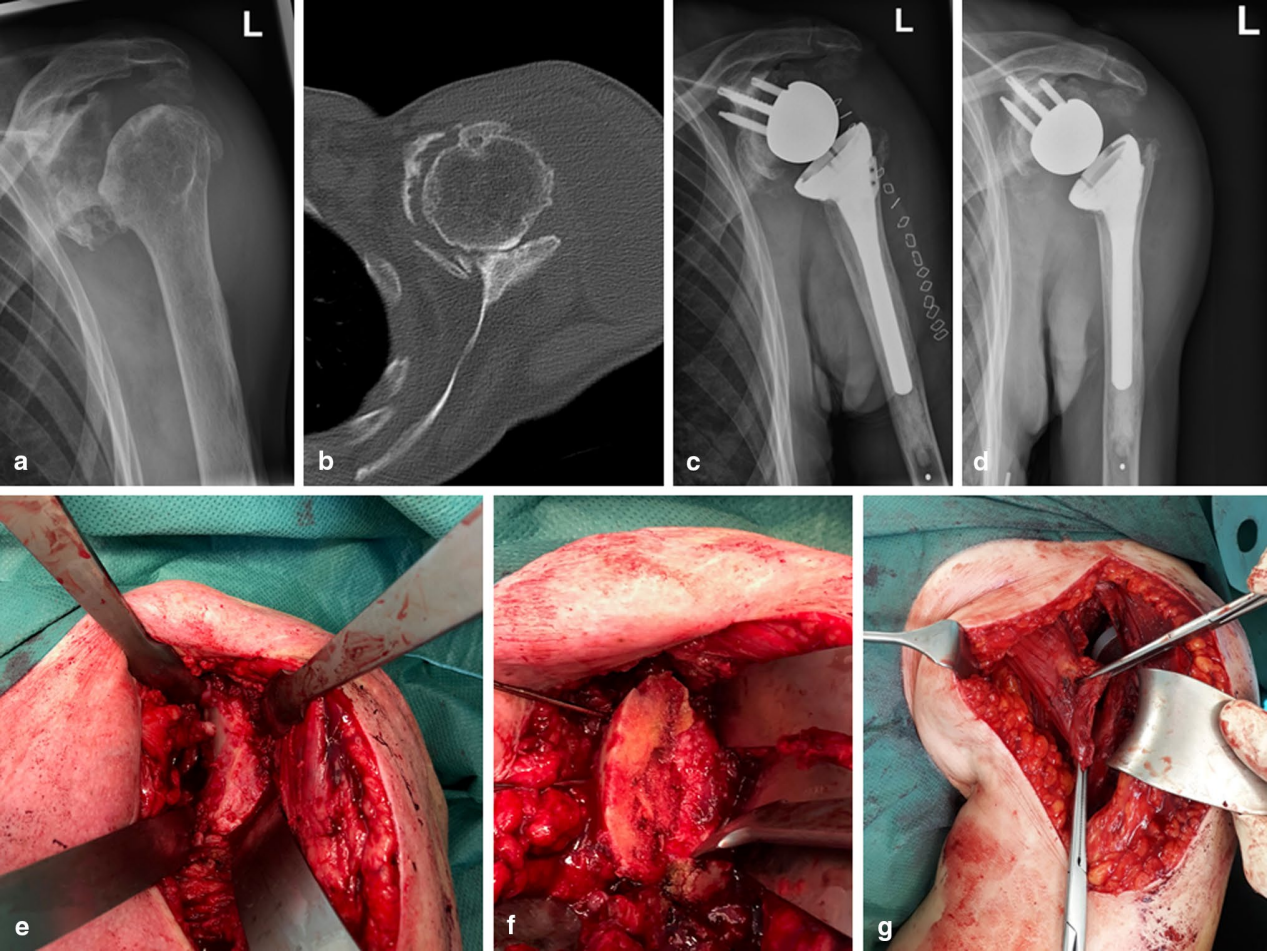
Preoperative (**a**, **b**), postoperative (**c**), follow-up images (**d**) and intraoperative images (**e**–**g**) of a 79-year-old female patient with a FS type 2c, i.e., a locked anterior dislocation with concomitant anterior glenoid bone loss of around 80% (**b**) treated with primary reverse shoulder arthroplasty with glenoid bone grafting with the resected humeral head (**e**, **f**) and pectoralis major tendon transfer for better soft tissue balancing (**g**). At final follow-up after 19 months, the Constant score was 75 points

For subtype 2 lesions of our suggested modified classification (Fig. 1), two different implant systems were used. For patients with a glenoid loss up to 40 percent, we used our standard reverse prosthesis (Univers Revers; Arthrex, Naples, USA). If the glenoid loss was greater 40 percent, we preferred to use a reverse prosthesis with a larger central screw (Altivate Reverse Shoulder Prosthesis; DJO; Dallas; USA), which also allows 135° humeral inclination. Tenotomy of the long head of the biceps was routinely performed. After exposition and resection of the humeral head, a baseplate was placed on the glenoid. If the glenoid bone loss was greater 30 percent, a glenoid augmentation was performed using the resected humeral head, i.e., an autologous bone graft, which was fixated either with the screws of the baseplate or with separate 3.0 mm cannulated CCS SpeedTip screws (Medartis; Basel; Switzerland). In all cases, a lateralized glenosphere was selected. The humeral stem was cemented in 2 cases (33%) and placed in a press-fit fashion in 4 cases (67%). In all cases, the humeral inclination was 135°. Following fixation of the humeral stem, the pectoralis major tendon was dissected from its humeral insertion, released and fixed to the lesser tuberosity transosseously. Exemplary images of the surgical technique are shown in Fig. 2.

### Aftercare

Postoperatively the shoulder was usually immobilized in internal rotation for 6 weeks. Passive range of motion (ROM) was initiated at 3 weeks postoperative. The sling was removed at 6 weeks and active range of motion was allowed. Strengthening was allowed at 12 weeks postoperative. In case of glenoid bone grafting, the shoulder was strictly immobilized in a sling for 6 weeks in order to minimize the stress on the graft and passive ROM was initiated afterwards.

### Postoperative evaluation

Data concerning characteristics of the patient at the moment of surgery, surgical technique, and complications were retrospectively retrieved from our institution’s electronic medical record system.

An independent observer (blinded to the performed procedure) examined patients and assessed the clinical outcome. For follow-up examination, the patients were asked to grade pain on a visual analog scale (VAS). Active ROM was measured with a goniometer for elevation, abduction, and external rotation of the elbow at the side. Internal rotation was judged by the level of vertebra reached by the thumb. Functional outcome was assessed using the Constant-Murley score (CS) as shoulder specific score. In addition, the subjective shoulder value (SSV) and the Quick Disabilities of Shoulder and Hand Score (DASH) were used as patient-focused outcome tools. In order to evaluate the patients’ general health condition, the VAS Eq5d score was used.

Radiographic assessment at follow-up was based on an AP view in neutral rotation as well as a Y-view and was performed by one examiner (JS). Scapular notching was evaluated in the AP view and classified according to Sirveaux [11].

#### Statistical analysis

Statistical analysis was performed with SPSS version 22 (IBM, Armonk, USA) using the independent samples Mann–Whitney *U*-test and the Kruskal–Wallis test. Quantitative variables were described by means, standard deviations, minimums and maximums. Normal distributions were tested by the Shapiro–Wilk test and confirmed graphically by histogram.

## Results

Of 69 patients with a FS of the proximal humerus, 26 presented a FS type 2. 16 patients (62%) with a mean age of 75 ± 6 years [range 61–83 years] were available for follow-up at 24 ± 18 months. 6 patients were deceased, 2 had to be excluded due to severe neurologic comorbidities and 2 refused to participate in the study. Baseline characteristics are summarized in Table 1. Patient outcomes throughout the whole cohort and subdivided into the three subgroups are outlined in Table 2. Of the type 2a lesions, 2 were treated with stemless total shoulder arthroplasty and 1 with stemless hemiarthroplasty. All type 2b lesions as well as all type 2c lesions were treated with reverse shoulder arthroplasty. For the type 2c lesions, glenoid bone grafting was performed in all cases with the resected humeral head and a pectoralis major tendon transfer was performed.

**Table 1:** Baseline characteristics

Variable	Number
Follow-up rate [percent]	16/26 [62%]
Mean patient age in years [SD]	75 [± 6]
Mean follow-up in months [SD]	34 [± 5]
Gender	
Male [percent]	2 [12%]
Female [percent]	14 [88%]
Injured side	
Right [percent]	10 [63%]
Left [percent]	6 [37%]
Subtype of fracture sequelae type 2	
Locked posterior (2a) [percent]	3 [19%]
Locker anterior (2b) [percent]	7 [44%]
Locked anterior with glenoid bone loss (2c) [percent]	6 [37%]

*SD* standard deviation

**Table 2:** Patient outcomes throughout the whole cohort and subdivided into the three subgroups

	All FS 2	Subtype 2a	Subtype 2b	Subtype 2c
Mean preoperative CS [points]	10 ± 6	14 ± 5	11 ± 7	7 ± 1
Mean postoperative CS [points]	58 ± 21	61 ± 25	60 ± 15	55 ± 27
Mean VAS [points]	2 ± 2	2 ± 3	2 ± 3	3 ± 2
Mean SSV [percent]	58 ± 23	73 ± 19	53 ± 14	55 ± 29
Mean Quick DASH [points]	27 ± 23	13 ± 17	33 ± 25	27 ± 23
Mean Eq5d VAS [percent]	58 ± 28	75 ± 13	45 ± 26	61 ± 22
Mean flexion [degrees]	110 ± 31	110 ± 27	123 ± 23	97 ± 35
Mean abduction [degrees]	101 ± 31	110 ± 45	98 ± 22	100 ± 32
Mean external rotation [degrees]	38 ± 15	57 ± 10	38 ± 13	28 ± 11
Mean internal rotation [vertebra level]	L2	L3	L4	L1

*CS* constant score, *DASH* disabilities of shoulder and hand, *EQ5D* European quality of life 5 dimensions, *FS* fracture sequelae, *L* lumbar vertebra, *SSV* subjective shoulder value, *VAS* visual analog scale

Mean postoperative active forward flexion was 110° ± 31°, mean abduction 101° ± 31° and mean external rotation at the side was 38° ± 15°. Mean internal rotation was at vertebra L4. The mean adapted CS was 71 ± 26 percent and the mean SSV was 58 ± 23 percent. Average pain level on the VAS was 2 ± 2 out of 10 points. The mean Eq5d general health score was 58 ± 28 percent. There were no statistically significant differences regarding the various outcome parameters between the three subgroups.

Preoperative CS improved significantly from 10 ± 6 points to 58 ± 21 points (*p* < 0.00001). The mean Quick DASH Score was 27 ± 23 and the mean SSV was 58 ± 23 points.

The complication rate was 19% and the revision rate was 6%; implant survival was 94%. In 1 case, an acromion insufficiency fracture occurred 6 months postoperatively and was treated conservatively with immobilization in an abduction pillow for 6 weeks. Another patient suffered an early infection with Staphylococcus aureus which was treated surgically by changing the mobile parts followed by 12 weeks of antibiotic treatment. In another case, a CT scan was indicated due to prolonged postoperative pain and both bone graft and glenoid component healed in a 70° anterior-medial malposition; however, the patient refused a surgical revision. The 3 patients who suffered a complication showed significantly inferior functional results compared to the rest of the cohort (mean CS 29 vs. 68 points; *p* = 0.003).

Glenoid bone loss for subtype 2c was measured according to the technique of Baudi et al. [12] and averaged 55% (range 30% to 80%).

Scapular notching could be observed in 2 (13%) cases (all grade 1).

## Discussion

Locked dislocations of the glenohumeral joint are disabling and often painful conditions resulting in very limited ROM and thus severely compromising the patients’ everyday life.

Treatment of chronic anterior glenohumeral dislocation with glenoid bone loss and concomitant rotator cuff deficiency remains a technical challenge. Persistent anterior dislocation often results in excessive wear of the anterior-inferior glenoid rim, humeral head deformity, and degenerative changes of the subscapularis muscle such as heterotopic ossification, tendon retraction and fatty degeneration. The combination of substantial glenoid bone loss, extensive periarticular soft tissue scarring, distension of the posterior joint capsule, and unbalanced retraction of the rotator cuff complicate the reconstruction of a stable glenohumeral articulation [13, 14].

Fracture sequelae of the proximal humerus are rare pathologies and were first classified by Boileau et al. [3] In his first case, series of 71 patients treated in a 5-year time period, only 9 fracture sequelae type 2 were described, whereas 8 were locked posterior dislocations. In 2006, Boileau et al. [4] published a multicenter study of 203 fracture sequelae of which 25 cases were classified as type 2. They were either treated with HA or TSA. All in all, CS improved from 28 preoperatively to 61 points at the final follow-up. Interestingly, for type 2 FS, the complication (32%) and revision rate (24%) were the highest compared to the other types of FS; however, these were not specified. Nevertheless, Boileau et al. recommended the treatment of type 2 FS with HA or TSA. In a cohort of 10 consecutive patients, Raiss et al. [2] reported an improvement of the preoperative CS from 20 to 60 points at the final follow-up after treatment with HA or TSA. The complication rate was 20% with 1 anterior dislocation postoperatively requiring revision surgery and 1 case of glenoid erosion. Unfortunately, glenoid bone loss was not quantified and glenoid morphology was only classified according to Walch [15] (6 type A1, 2 type A2, 2 type C). Matsoukis et al. [1] described a series of 11 patients with fixed anterior glenohumeral dislocation treated with HA or TSA. The authors reported an age and gender adjusted Constant score of 60% 48 months postoperatively. Seven complications in five shoulders (45%) were observed, in four cases recurrent anterior dislocation occurred. Glenoid bone loss was judged intraoperatively by the surgeon and was greater than one-third of the anterior-to-posterior diameter of the glenoid in four patients. Due to the unpredictable results and the high rate of recurrent instability after HA or TSA, in the near past, RSA with its semi-constrained concept has been increasingly used for the treatment of type 2 FS, especially in cases with glenoid bone loss [6, 16].

In 2014, Werner et al. [16] published a series of 21 patients with chronic anterior shoulder dislocation and concomitant glenoid bone defect treated with RSA. The CS increased from 6 to 57 points at the final follow-up. Bone grafting was performed in all cases, whereas only 2 patients had a glenoid failure. One case of glenoid loosening was related to trauma and the other to inadequate fixation of the central peg in the native bone. They concluded that the central peg should be inserted at least 10 mm into the native bone and that the baseplate should be seated on minimum 50% native bone. Therefore, they stated that exact preoperative CT planning is obligatory and that baseplate components with long pegs (25 mm) should be available. Raiss et al. [6] published a multicenter study of 22 patients with chronic locked shoulder dislocation treated with RSA. The CS increased significantly from 14 to 47 points at final follow-up. There were 7 complications (32%), leading to revision surgery in 6 cases. Failure of the glenoid component occurred in 4 cases. After glenoid bone grafting, the failure rate was 80%. This could either be due to technical mistakes such as insufficient graft fixation (central pegs with only 15 mm of length were used) or malposition of the graft (not covering sufficient native bone). Another explanation could be that the soft tissue adaptations in a chronic dislocated shoulder joint were underestimated. Before starting our case series, we saw several cases referred to our clinic due to a dislocated reverse prosthesis after treating chronic anterior locked dislocations with RSA and during revision surgery we realized that the problem was the retracted and in some cases ossified subscapularis tendon pulling the humeral shaft anteriorly.

The postoperative results in our cohort are comparable to those in the literature, especially considering the high percentage of patients with a chronic anterior dislocation with glenoid bone loss. Furthermore, the revision rate was very low on the short-term.

**Fig. 3:**
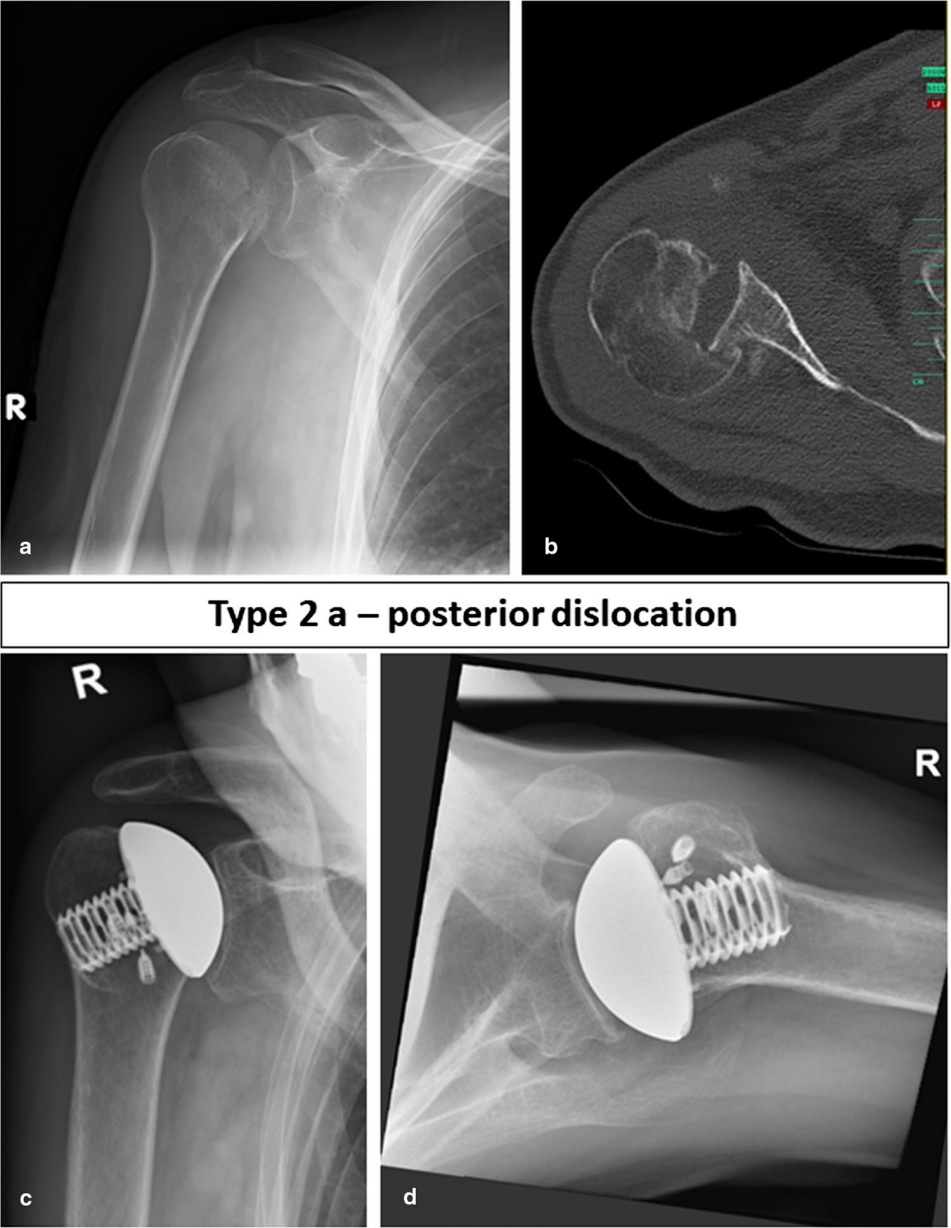
Preoperative (**a**, **b**) and follow-up images (**c**, **d**) of a 76-year-old female patient with a FS type 2a, i.e., a posterior dislocation treated with primary anatomic hemiarthroplasty. At final follow-up after 12 months, the Constant score was 70 points

**Fig. 4:**
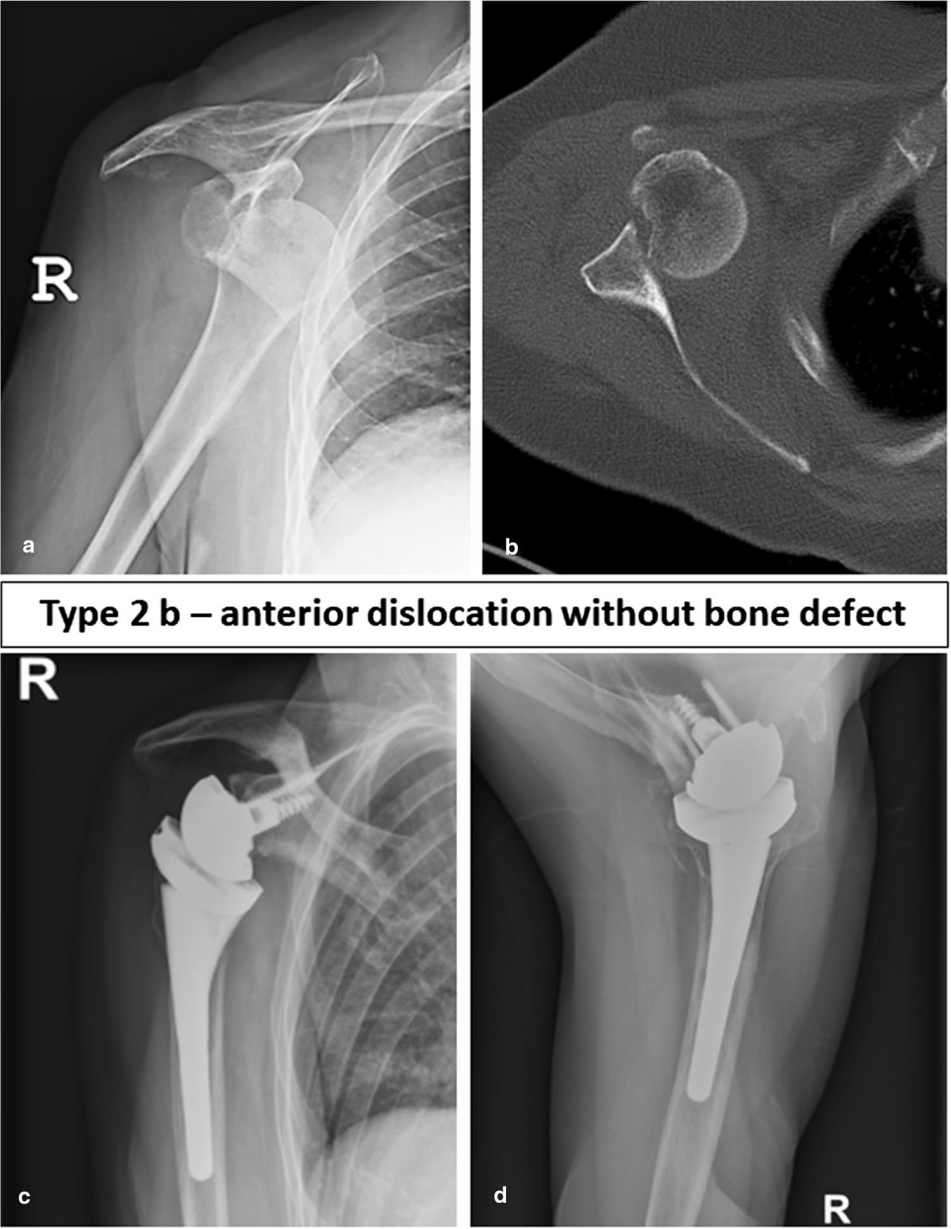
Preoperative (**a**, **b**) and follow-up images (**c**, **d**) of a 76-year-old female patient with a FS type 2b, i.e., an anterior dislocation treated with primary reverse shoulder arthroplasty. At final follow-up after 65 months, the Constant score was 71 points

**Fig. 5:**
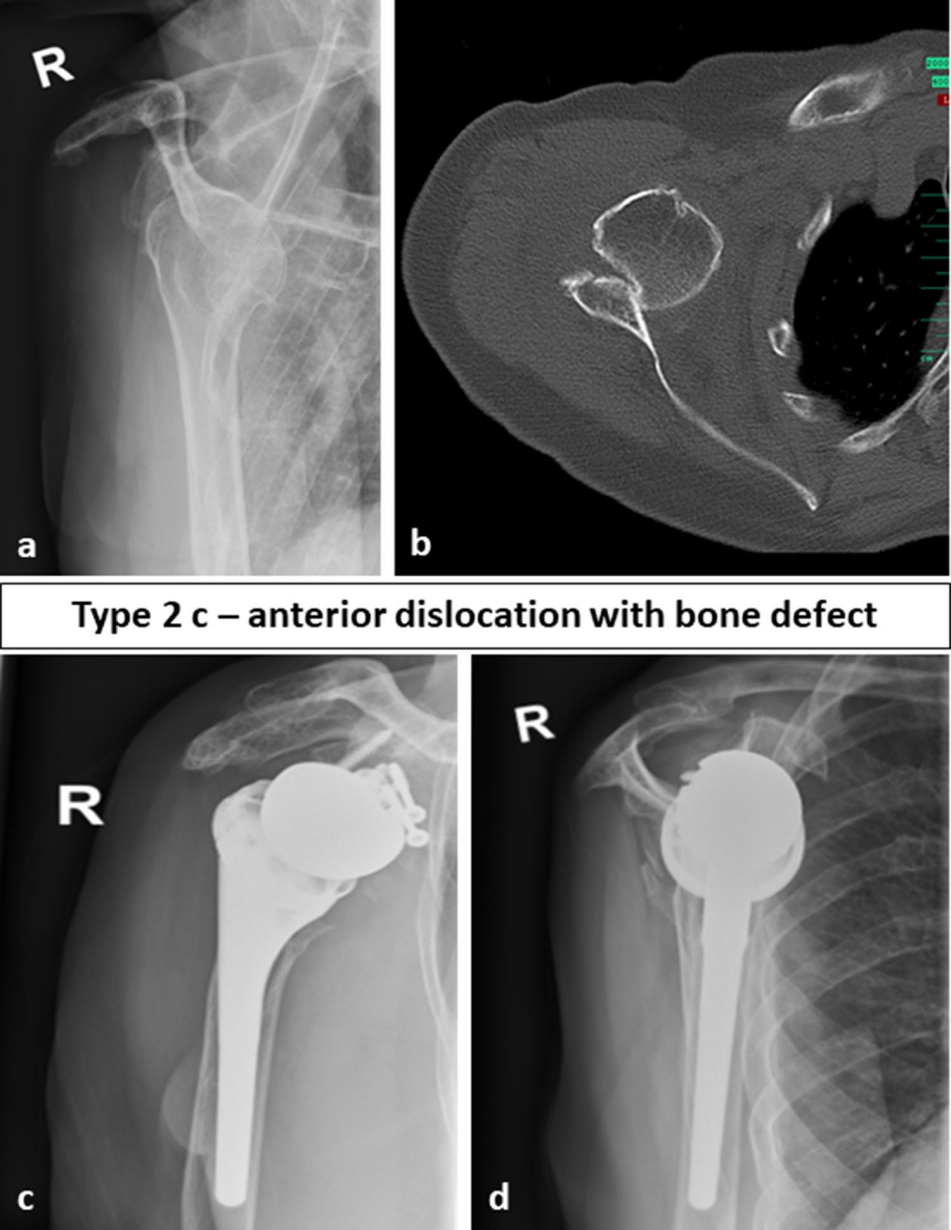
Preoperative (**a**, **b**) and follow-up images (**c**, **d**) of a 81-year-old female patient with a FS type 2c, i.e., an anterior dislocation treated with autologous glenoid bone grafting, primary reverse shoulder arthroplasty and pectoralis major transfer. At final follow-up after 32 months, the Constant score was 69 points

Thus, in our eyes, the classification of fracture sequelae according to Boileau is insufficient for type 2 lesions and the general treatment recommendation with HA or TSA has to be reconsidered. For type 2 lesions, glenoid bone loss has to be taken into account as this has a direct impact on the treatment. Therefore, in all type 2 FS, a preoperative computed tomography (CT) scan with a 3D reconstruction should be obtained to be able to quantify glenoid bone loss and to visualize the full extent of the joint deformity. Considering the current literature including our study, it is impossible to give an evidence-based recommendation in which cases RSA is indicated and at what extent of glenoid bone loss bone grafting should be performed. However, the mid-term data for the treatment of osteoarthritis and concomitant biconcave glenoid with TSA published by Walch et al. [17] should be considered and if anterior glenoid bone defects are comparable to reverse type B2, B3 or C defects according to Walch [15] RSA should be performed as the complication rate is reported to be very high for TSA in mid- to long-term results [17]. We suggest the subclassification of the type 2 fracture sequelae in three different subtypes. Locked posterior dislocations, i.e., type 2a (normally without glenoid defect) are suitable for HA or TSA. Chronic locked anterior dislocations (type 2b) should be treated with RSA and in cases with glenoid bone loss (type 2c) which is greater than 30 percent bone grafting should be performed with the resected humeral head. Exemplary cases are shown in the Fig. 3 (type 2a), 4 (type 2b) and 5 (type 2c) (Figs. 3, 4 and 5). In our case series, in cases with glenoid bone loss, the bone graft was either fixated with the screws inserted through the baseplate alone or in combination with cannulated compression screws with a diameter of 3.0 mm.

For long-standing anterior dislocations, the condition of the subscapularis muscle has to be evaluated during surgery. In case of chronic degeneration, a pectoralis major tendon transfer (PMT) should be considered to restore the force couple in order to secure stability and congruency of the implanted prosthesis. Applying these principles we achieved good clinical short-term results with a relatively low complication and a low revision rate compared to the current literature. Interestingly, in our cohort, no dislocation occurred and the patients in subgroup 2c who were treated with addition PMT showed a better internal rotation than the other two subgroups. We assume that this is due to the soft tissue management using a PMT to restore the force couple. However, as there are too many influencing factors a causal relation cannot be shown.

### Limitations

There are several limitations to our study. These clinical outcomes correspond to our initial experience, sample size is therefore small, and we report our short-term results. No complications or revisions of the patients who were lost to follow-up or deceased were documented. Further evaluation should be considered to confirm our recommendations.

## Conclusion

With good preoperative planning and by using the adequate surgical technique, good clinical short-term results with a relatively low complication rate can be achieved in cases with FS type 2. The authors suggest extending the Boileau classification for fracture sequelae type 2 and recommend using a modified classification to facilitate the choice of treatment as the suggested classification system includes locked posterior and anterior dislocations with and without glenoid bone loss. In chronic locked anterior dislocations with glenoid bone loss, RSA with glenoid bone grafting in combination with a pectoralis major tendon transfer should be considered as a feasible treatment option.

## Data Availability

Data are available on reasonable demand.
